# Healthy and Safe Swimming Week — May 20–26, 2019

**DOI:** 10.15585/mmwr.mm6819a1

**Published:** 2019-05-17

This year’s Healthy and Safe Swimming Week theme, “Pool Chemistry for Healthy and Safe Swimming,” focuses on preventing pool chemical injuries. Pool chemicals prevent the spread of germs that cause illnesses and disease outbreaks; however, these same chemicals can cause injuries if mishandled. Each year, an estimated 3,000–5,000 emergency department visits caused by pool chemical injuries (e.g., poisonings from inhalation or ingestion of pool chemicals and dermatitis or conjunctivitis from pool chemical splashes) occur in the United States.

This issue of *MMWR* includes a report focusing on pool chemical injuries leading to U.S. emergency department visits during 2008–2017 and a 2018 toxic chlorine gas incident in New York (*1*). Following product label directions, wearing proper safety equipment (e.g., respirator or goggles) when handling chemicals, and keeping chemicals out of the reach of children and teens can help prevent these injuries.

CDC’s Model Aquatic Health Code (MAHC; https://www.cdc.gov/mahc) is a set of recommendations that can be voluntarily adopted by state and local jurisdictions to help prevent pool chemical injuries, disease outbreaks, and drowning associated with public treated recreational water venues (e.g., pools, hot tubs/spas, and water playgrounds at hotels and apartment complexes). Swimmers can help by showering before getting in the water, never urinating or defecating in the water, and taking young swimmers on bathroom breaks or checking their diapers every hour.

CDC updates the MAHC every 3 years in coordination with the Council for the MAHC (https://www.cmahc.org/). CDC encourages public health officials to submit MAHC change requests to CMAHC by January 6, 2020, (https://www.cmahc.org/enter-change-request.php) to be considered for the 2021 MAHC (4th edition).
